# Psychogenic fever in a patient with small cell lung cancer: a case report

**DOI:** 10.1186/s12885-015-1462-z

**Published:** 2015-05-30

**Authors:** Mengdan Xu, Xiaoye Zhang, Zhaoguo Xu, Guoyuan Cui, Li Yu, Xiaoying Qi, Jia Lin, Yan Liu

**Affiliations:** Department of Oncology, Shengjing Hospital of China Medical University, Shenyang, 110004 China

**Keywords:** Psychogenic fever, Anxiety, Small cell carcinoma, Duloxetine

## Abstract

**Background:**

Fever is common in malignant tumors. We report an exceptional case of psychogenic fever in a patient with small cell lung cancer. This is the first case report of psychogenic fever in a patient with small cell lung cancer.

**Case presentation:**

A 61-year-old Chinese man diagnosed with small cell carcinoma on June 30, 2012, came to our department with a complaint of fever lasting more than 1 month. He had undergone chemoradiotherapy for lung cancer 6 months previously. After admission, his body temperature fluctuated in the range of 37 °C to 39 °C. Somatic symptoms associated with anxiety were obvious. A 24-item Hamilton Anxiety Scale was used to assess the patient’s condition. A score of 32 confirmed a diagnosis of severe anxiety. After a week of antianxiety treatment, the patient’s temperature returned to normal.

**Conclusion:**

Psychogenic fever is common in cancer patients and deserves more attention. Patients with psychogenic fever must be distinguished from patients with infectious fever (including neutropenic fever), and tumor fever. Additionally, antianxiety or antidepression treatment should be provided. A concern is that continual anxiety may adversely affect anticancer therapy.

## Background

Psychogenic fever is higher than normal body temperature during conditions of acute or persistent psychological stress. Psychogenic fever has been reported in patients 3–56 years old with a sex ratio of 1:1.19 (male vs female) [1]. Many studies have demonstrated that psychological stress affects core body temperature in laboratory animals, with acute stress inducing transient hyperthermia [2, 3] and repeated, chronic stress inducing persistent low-grade hyperthermia [4, 5], which facilitates a hyperthermic response to novel stressors [6]. Animal studies have suggested that the presence of β-endorphin, a corticotropin-releasing hormone, and noradrenaline within the brain, along with activation of the sympathetic nervous system, are involved in acute stress-induced hyperthermia [7]. Habitual hyperthermia in young women, many of whom are under treatment for neuroses, has attracted increasing interest in recent years. In some cases anxiety or tension seems to be responsible, and expression of the problem, together with resolution of difficulties causing the stress, has resulted in defervescence [8]. Such mechanisms may have contributed to the increase in core temperature (Tc) in our patient. Pyrogenic cytokines induced by stress may increase Tc in patients with psychogenic fever. However, to date, little is known about how psychological stress elevates Tc in patients with psychogenic fever or in otherwise healthy subjects.

Patients with lung cancer, especially in an advanced stage, often manifest the symptom of fever. The most common causes for this are infection (including those associated with neutropenic fever due to radiotherapy and/or chemotherapy), followed by tumor fever induced by tumor progression or high tumor burden. Cancer is often accompanied by anxiety and depression, which results in psychogenic fever.

Though not commonly reported, psychogenic fever is one of the symptoms of anxiety and depression. To our knowledge, psychogenic fever has not been reported in patients with cancer, but we encountered what we believe to be a case in our clinical practice that is of interest. After receiving treatment with antianxiety agents, both the elevated body temperature and anxiety were significantly alleviated.

## Case presentation

### Case report

The patient was a married 61-year-old male. On June 21, 2012, a computed tomography (CT) scan of the chest showed a left lung space-occupying lesion and small cell carcinoma that was subsequently confirmed by bronchoscopic biopsy. His TNM classification was T2N1M0 with clinical staging IIB. After completion of four cycles of chemotherapy consisting of etoposide plus cisplatin, he underwent one course of concomitant lung radiotherapy treatment of unknown radiation dose. After chemoradiotherapy the cancer was in complete remission as of Octobor 2012. In November 2012, the patient developed fever without obvious causes, with a temperature fluctuation between 37.5 °C and 39.8 °C, with no chills or shivering, no cough, no sputum, no abdominal pain or sore throat, no urinary frequency or urgency, or dysuria. The patient successively received intravenous infusions of cefoxitin, moxifloxacin, meropenem, fluconazole and vancomycin for anti-infection treatment in another hospital, but his temperature did not improve.

**Fig. 1:**
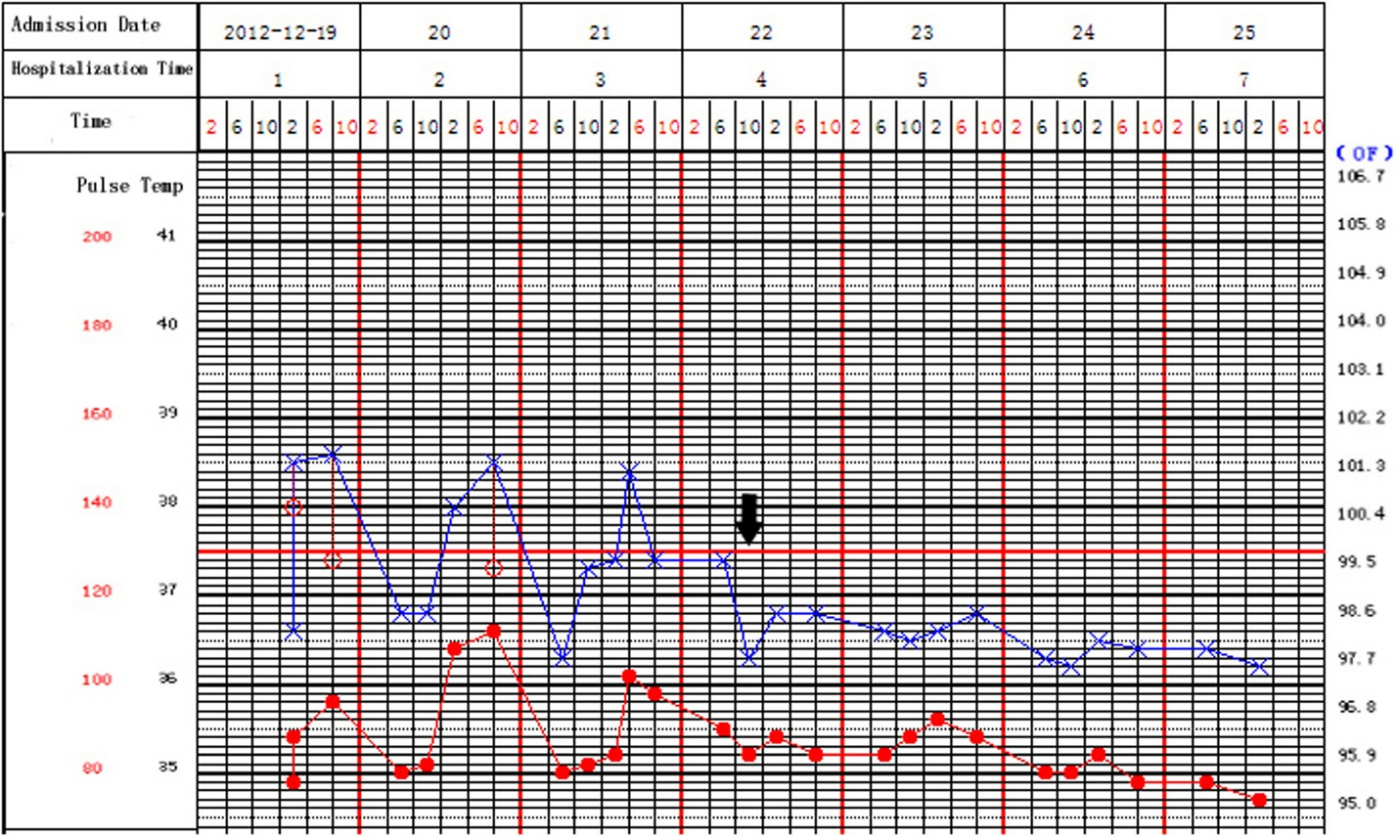
Clinical course. Blue lines and X-marks shows axillary temperature; red lines and circles mean changes of heart rate. Axillary temperature was measured 4 times daily: at 6:00 a.m.; 10:00 a.m.; 2:00 p.m.; and 8:00 p.m. (blue line). In the process of his intermittent fever ,his temperature spontaneously fell down,and he did not take any antipyretics. After anti-anxiety treatment, the axillary temperature of the patient returned to normal level (black arrow)

On December 19, 2012, the patient was admitted to our department with a complaint of fever lasting for more than 1 month. He had undergone chemoradiotherapy for lung cancer 6 months previously. After admission, the patient experienced intermittent fever, and his body temperature fluctuated in the range of 37 °C to 39 °C (Fig. 1). There were no chills, shivering, cough or expectoration, but the patient had disordered sleep, facial flushing, an anxious facial expression, chest discomfort, palpitation and shortness of breath. He seemed agitated and spoke very quickly with stuttering, and had frequent nocturnal urination (5 times/night). The patient denied any history of tuberculosis, hepatitis or other infectious diseases, and had no family history of hypertension, diabetes or tumor.

Physical examination showed: temperature 38.2 °C, respiratory rate 22 breaths per minute, blood pressure 130/85 mm Hg, and heart rate 115 beats/min. There was no skin redness, swelling burning sensation, or pain. His eyelids quivered when closed. His hands shook slightly and there was slight redness of the pharynx. His tonsils were not enlarged. He had neither dry nor wet rales. The heart sound was clear without murmur and with a normal heart rhythm. There was no abdominal tenderness or rebound pain, no percussive pain in the liver or kidney area, and no tenderness in the gall bladder or appendix area.

**Fig. 2:**
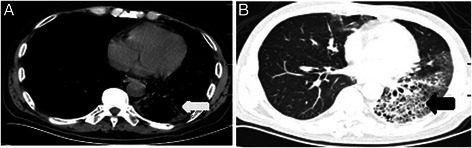
CT scan results. Computed tomography scan of the chest taken post-radiotherapy on December 21, 2012 (large arrow in **a** and **b**) showing: central type cancer of the left lung, radiation pulmonary fibrosis

Blood and urine biochemical indices were all within a normal range. The result of a 1-3-beta-glucan test was less than 10 pg/mL. Other test results were: white blood cell count: 9.4*10^9^/L; neutrophils 72.5 %; (twice), C-reactive protein < 3.300 mg/L; fasting plasma glucose: 5.4 mmoL/L; hepatitis virus was negative. CT showed no calcification segmentation. Pulmonary CT was performed in December 20, 2012 and signs of pulmonary interstitial fibrosis were noted in the left lower lobe and the medial segment of the right middle lobe of the lungs (Fig. 2).

In consultation with physicians from the psychology department in our hospital, a 24- item Hamilton Anxiety Scale was used to assess the patient’s condition. A score of 32 confirmed a diagnosis of severe anxiety. Of all the items on that scale, the scores for the sub-items insomnia, anxious mood and depressive mood were among the top 3 ratings (3, 4, and 4, respectively), and corresponding somatic symptoms were obvious: under assessment, the patient was nervous with his hands clasped, while his facial muscles clearly twitched, showing an obvious pallor and stiff face.

A previous combination of antibiotics had no effect. The patient had a history of radiation therapy, so fibrotic changes in the pulmonary interstitium were considered radiation-induced pulmonary fibrosis. The clinical diagnosis was severe anxiety, psychogenic fever; right central-type lung cancer; and radiation-induced pulmonary fibrosis.

The treatment course consisted of duloxetine (Eli Lilly and Company) 30 mg/day, clonazepam (Nhwa Pharma. Corp.) 0.5 mg/day; 4 days later, the duloxetine dose was increased to 60 mg/day and the clonazepam dose was increased to 1.0 mg/day. After 1 week, the patient’s temperature returned to normal (Fig. 1) and his sleep disorder resolved. He was without facial flushing and the symptoms such as chest discomfort and shortness of breath also improved. The palpitations and agitation resolved, his speech rate became normal and stuttering stopped. The quivering in his eyelid was significantly alleviated and the hand trembling disappeared. Nocturnal urinary frequency improved and his anxiety was significantly improved. The Hamilton Anxiety Scale retest score was 14, and the scores on the sub-items insomnia, anxious mood and depressive mood were 1, 2 and 1, respectively. Considering his condition, the patient was diagnosed with mild anxiety and discharged. After discharge, a brain metastasis was found at a local hospital in April 2013, but he did not accept further treatment and subsequently died in June 2013. However, the psychogenic fever did not recur during this period.

## Discussion

The differential diagnosis of psychogenic fever is of great importance. In general, infectious fever should first be excluded. In our patient, routine blood tests performed multiple times were within normal ranges, percussive pain was negative in the renal region, and urinary tract infection was excluded. The patient had received antifungal (fluconazole) therapy in another hospital without result so fungal infection was excluded. The patient had no abdominal distension or pain, and whole abdomen tenderness, rebound tenderness and Murphy’s sign were all negative. Appendix area tenderness was also negative, thus peritonitis, cholecystitis and appendicitis were excluded. No manifestations of infection such as superficial skin redness, swelling, burning sensation or pain were observed. The blood culture was negative. Given the lack of evidence of infection or toxic symptoms, fever induced by a skin infection or sepsis was excluded. CT showed fibrotic imaging changes in the pulmonary interstitium in the left lower lobe and medial segment of the right middle lobe, which were differentiated from atypical pneumonia. Because *Mycoplasma pneumoniae*, *Chlamydia pneumoniae* and *Legionella* antibodies were all negative, and the patient had once received radiotherapy, as indicated by obvious honeycombing in his inferior lobe of left lung, atypical pneumonia was excluded and radiation-induced pulmonary fibrosis was diagnosed. The mainstay treatment for radiation pneumonitis is oral steroid treatment. No steroid treatment was given during the course of the disease. Because the antianxiety agents improved the fever in a short time, we rejected the possibility of fever caused by radiation pneumonitis. In addition, the patient had once received an intravenous infusion of cefoxitin, moxifloxacin, meropenem and vancomycin for anti-infection treatment in another hospital, at which time his temperature showed no improvement, thus excluding the possibility of infectious fever.

Psychogenic fever should not be confused with febrile episodes deliberately contrived by manic patients, which represents a variant of Munchausen syndrome [9]. In patients with cancer, stress-induced fever should be distinguished from tumor fever. Tumor fever is often induced by tumor load or rapid proliferation. Other mechanisms include tumor necrosis, which may be associated with the release of tumor necrosis factors and other pyrogens from dead tissue [10]. The determination of complete remission following chemotherapy and radiotherapy also did not support the diagnosis of tumor fever.

Psychogenic fever is very common in patients with anxiety and depression. Activation of the sympathetic nervous system is known to increase core body temperature by increasing thermogenesis, including non-shivering thermogenesis in brown adipose tissue, and by decreasing heat loss with peripheral vasoconstriction [7]. Because this patient’s anxiety was obvious, a Hamilton Anxiety Scale assessment was carried out in consultation with physicians from the psychology department of our hospital, who diagnosed severe anxiety. After treatment with antianxiety agents, the patient’s temperature quickly returned to normal, which further confirmed the diagnosis of psychogenic fever. In this case, fever onset occurred about 1 month after chemoradiotherapy, so we do not think the fever was associated with chemoradiotherapy. We think the cause of psychogenic fever in this case was his fear of a malignant tumor. Fear of the malignant tumor caused mental stress, leading to a series of pathophysiological changes that caused fever.

Once psychogenic fever is diagnosed, prompt treatment should be provided. In this case, the patient received the combination of duloxetine and clonazepam for antianxiety treatment, which demonstrates a better synergistic effect and faster function than either drug alone.

## Conclusion

The high incidence of anxiety in cancer patients is often accompanied by complex physical symptoms that are easily overlooked or confused with other conditions. Somatic symptoms associated with anxiety have seriously affected the quality of life of cancer patients and even patient compliance with anticancer treatment. Although psychogenic fever is not common in cancer patients, it should be considered when other causes are not apparent. Patients with psychogenic fever must be distinguished from patients with infectious fever (including neutropenic fever) and tumor fever. Additionally, antianxiety or anti-depression treatments should be provided to avoid interference with anticancer therapies.

## Consent

Written informed consent was obtained from the patient for publication of this case report and any accompanying images. A copy of the written consent is available for review by the editor of this journal.

## Abbreviations

Tc: Core temperature
CT: Computed tomography
